# Analysis of Health Care Providers' Attitudes and Expectations on Intradialytic Exergaming: Qualitative SWOT Study

**DOI:** 10.2196/106283

**Published:** 2026-11-04

**Authors:** Aurel Zelko, Marianna Antoniadou, Jaroslav Rosenberger, Zuzana Dankulincova Veselska, Andrea Madarasova Geckova, Leonie Klompstra, Tiny Jaarsma

**Affiliations:** 1 Department of Health Psychology and Research Methodology, Faculty of Medicine, University of Pavol Jozef Šafárik, Trieda SNP 1, Košice, 040 11, Slovakia; 2 Department of Health, Medicine and Caring Sciences, Linköping University, Växthuset, House 511-001, Linköping, Östergötland, SE-581 83, Sweden, 46 11363550; 3 Department of Community and Occupational Medicine, University Medical Center Groningen, University of Groningen, Groningen, The Netherlands; 4 Olomouc University Social Health Institute, Palacký University Olomouc, Olomouc, Czech Republic; 5 Department of Internal Medicine, Faculty of Medicine, University of Pavol Jozef Šafárik, Košice, Slovakia; 6 Fresenius Medical Care Dialysis Services Košice, Košice, Slovakia; 7 Institute of Applied Psychology, Faculty of Social and Economic Sciences, Comenius University Bratislava, Bratislava, Slovakia

**Keywords:** hemodialysis, exergaming, qualitative research, intradialytic exergaming, health care providers, physical activity

## Abstract

**Background:**

Physical inactivity significantly contributes to the rapid decline in clinical and health-related outcomes among patients undergoing hemodialysis. Exergaming may offer a safe, effective, and sustainable solution for maintaining adequate physical activity levels in these patients.

**Objective:**

This study aims to support its clinical applicability; our aim was to examine the attitudes and expectations of health care providers regarding intradialytic exergaming (IEG) in hemodialysis care in Slovakia.

**Methods:**

We conducted 21 individual, in-person, semistructured interviews with health care providers. Using purposive sampling, we included training assistants, nurses, nephrologists, and managers from 2 dialysis centers in Slovakia. The verbatim transcripts of interviews were qualitatively analyzed using an inductive thematic analysis approach. The identified codes and themes were further deductively categorized into the strengths, weaknesses, opportunities, and/or threats quadrants of the Strengths, Weaknesses, Opportunities, and Threats (SWOT) framework.

**Results:**

We identified 2 strengths, 2 weaknesses, 3 opportunities, and 3 threats of IEG. Strengths included health care providers’ enthusiasm and belief in the safety of IEG. Weaknesses involved concerns about the complexity of IEG and low digital literacy among patients and providers. Opportunities were seen in improving information availability and specificity, adjusting clinical culture, and tailoring exercises. Threats included skepticism about the availability of financial resources for IEG, resistance to changes in clinical practice, and issues related to patient behavior and privacy.

**Conclusions:**

Health care providers perceived IEG as a safe and appropriate intervention for clinical practice. To improve implementation strategies, it is important to consider specific expectations regarding resource availability, as well as the technological and organizational aspects of IEG.

## Introduction

Maintaining adequate physical activity is one of the key challenges in the self-management of patients with chronic conditions. Kidney Disease Improving Global Outcomes Clinical Practice Guideline for the evaluation and management of chronic kidney disease recommends that all patients with chronic kidney disease be advised to undertake moderate-intensity physical activity for a cumulative duration of at least 150 minutes per week, or to a level compatible with their cardiovascular and physical tolerance [1]. Insufficient physical activity in patients with chronic diseases is associated with higher rates of hospitalization, disability, and mortality, as well as increased financial and clinical burdens on both individuals and the health care system [2-5]. Health-related complications and behavioral limitations associated with primary chronic diseases, coexisting comorbidities, and the side effects of therapeutic procedures negatively affect patients’ physical activity levels [6-11]. To address these complications and limitations, various exergaming strategies have been introduced as innovative behavioral interventions aimed at reducing sedentary lifestyles and promoting physical activity among individuals with chronic diseases [12-18].

Current evidence on the clinical application of exergaming, defined as the interactive experience of playing video games that require physical exertion or movement [19], indicates beneficial effects on physical activity behaviors and overall physical functioning in patients with chronic diseases. Benefits include greater adherence to recommended daily physical activity levels, enhanced muscle strength, endurance, physical capacity, and improved postural control, balance, and motor functions [20-27]. Maintaining regular physical activity, muscle function, and overall physical conditioning is a fundamental aspect in clinical management of chronic kidney disease, particularly for patients undergoing hemodialysis [28-30]. Low levels of physical activity and their progressive decline over time in patients undergoing hemodialysis are associated with increased morbidity, higher risks of all-cause and cardiovascular mortality, and poorer disease prognosis [31-36]. Given that many of these patients experience limitations and insufficiency in performance of daily physical activity and face a high risk of protein-energy wasting, malnutrition, and dynapenia or sarcopenia [37-41], intradialytic exergaming (IEG) may serve as a well-suited and effective component of clinical behavioral interventions [42-45].

The development and implementation of clinical behavioral interventions that primarily target physical activity and exercise through exergaming technologies present a highly challenging task [46-48]. Planning, execution, and continuation of such interventions for patients undergoing hemodialysis demand a comprehensive understanding of the disease’s clinical and therapeutic background, the physiological effects of exercise, and current clinical guidelines for exercise prescription [28,49]. Additionally, effective integration of technological solutions into clinical practice requires consideration of logistical, financial, legislative, and organizational factors [50,51]. From an organizational perspective, it is necessary to understand and consider the qualitative dimensions of patients’ and health care providers’ awareness, knowledge, expectations, skills, competencies, cognition, and attitudes toward IEG [52-57].

Therefore, the aim of our study was to explore attitudes and expectations of health care providers regarding IEG in hemodialysis care in Slovakia. As participating health care providers had no prior experience with IEG, our study did not seek to evaluate actual experiences with the intervention. Rather, it focused on health care providers’ hypothetical perceptions, attitudes, and expectations concerning the potential implementation of IEG in hemodialysis care. To achieve this objective, we used the Strengths, Weaknesses, Opportunities, and Threats (SWOT) framework to analyze collected data and guide the formulation of results. The SWOT framework provides a practical approach for evaluating the factors that may influence the success or failure of a procedure by examining both internal capabilities (Strengths and Weaknesses) and external challenges (Opportunities and Threats). It organizes these factors into 4 categories: Strengths are internal assets that enhance performance indicators and support the achievement of objectives. Weaknesses refer to internal limitations that may reduce the acceptability or effectiveness of the procedure. Opportunities are external conditions or emerging trends that can be leveraged to improve outcomes or broaden impact. Threats encompass external risks or challenges that may undermine the procedure’s success or sustainability. As a strategic management tool, SWOT analysis supports informed decision-making by identifying key internal and external factors, thereby facilitating the effective introduction and integration of innovative procedures into clinical practice [58-62].

## Methods

### Study Design

This study was part of a project investigating the acceptability and effectiveness of a 12-week progressive, individualized, supervised intradialytic resistance training intervention on muscle function among patients undergoing hemodialysis. The study was an ancillary qualitative substudy conducted within the overarching framework of project NCT03511924 (ClinicalTrials.gov) [63]. Although the intervention resulted in moderate to large improvements in muscle function, the acceptability of intradialytic exercise and the sustainability of its benefits remained low [64,65]. In the current study, we used a 2-step qualitative design to explore and categorize health care providers’ attitudes and expectations regarding IEG among patients undergoing hemodialysis. In the first step, we explored health care providers’ perspectives using inductive thematic analysis (guided by [66] of data collected from participants’ interviews. In the second step, we deductively classified the identified codes and themes using a SWOT analysis approach (as outlined [59].

### Participants

We recruited participants from 2 dialysis centers located in Slovakia. Training assistants, nurses, nephrologists, and managers were eligible to participate if they had been employed or actively involved at the dialysis center for the entire duration of the 3-month intradialytic exercise intervention and were at least 18 years of age. This criterion ensured that participants had direct experience with the implementation of intradialytic exercise in routine clinical practice and were able to provide hypothetical insights into factors influencing the initiation and implementation of IEG. Furthermore, nurses, nephrologists, and managers were required to have a minimum of 2 years of professional experience in their respective roles. All available health care providers from 2 dialysis centers were prescreened based on their age, sex, and professional experience, including the total number of years worked in both current and previous health care positions and roles. While all participants had experience with the previously implemented intradialytic exercise intervention, none had prior experience with IEG. To ensure diversity within the sample, individuals of different ages and professional backgrounds were purposefully selected and invited to participate in the study via an email sent by 2 members of the research team (AZ and IMS). Health care providers who accepted the invitation received oral and written information about the study, provided written informed consent, and had their interview date and time scheduled. We continued to include participants until we reached sufficiency of information for all studied topics. This occurred when responses to questions from successive participants became repetitive and no longer provided new, unique insights to the outcome of the study [67].

### Data Collection and Procedures

Individual, in-person, semistructured interviews with health care providers were conducted using an interview guide (Multimedia Appendix 1). The interviews began in June 2019, approximately 1 month after the completion of the intradialytic exercise intervention study. Members of the research team (AZ, IMS, and DK) developed the interview guide using the Patient-Centered Access to Health Care framework [68] and the Biopsychosocial Model of Responsiveness to Physical Activity [69] as conceptual frameworks. Three senior members of the research team (JR, ZDV, and AMG) evaluated the initial interview guide and proposed adjustments to enhance its clarity. Further minor adjustments were made following a pilot interview with one dialysis center health care provider. During the interview process, the researchers continually adjusted the guide to delve into areas of specific interest, aligning with best practices for qualitative data collection [70].

The final interview guide covered 4 scope areas (detailed in Multimedia Appendix 1). The first 3 areas explored (1) the perceived benefits of physical activity for dialysis patients in general, (2) the perceived benefits and risks of intradialytic exercise, and (3) experiences with implementing exercise interventions in clinical settings. Findings from these 3 areas were presented in a separate article [65]. The fourth area focused on participants’ perspectives on variations in intradialytic exercise interventions, with particular emphasis on IEG. Participants were asked about their opinions and suggestions on alternatives to intradialytic exercise interventions, as well as their attitudes and expectations regarding IEG. Data from this area served as the basis for the current article. The open-ended questions in the interview guide allowed participants to share their attitudes and expectations regarding IEG, enabling interviewers to probe deeper into individual perspectives. Participants were encouraged to elaborate and highlight issues they deemed important. The research team facilitated verbal expression and logical thought development, taking field notes when necessary.

Three members of the research team (AZ, IMS, and DK) conducted interviews from June 2019 through November 2019. Prior to the interviews, written informed consent was obtained from participants, along with a brief personal questionnaire summarizing their sociodemographic characteristics (age and sex) and professional experiences (years in previous and current job positions). The interviews were held in Slovak language, either in private meeting rooms at dialysis centers (for nurses and nephrologists) or in an office at the authors’ workplace (for assistants and management staff). All interviews were conducted individually, involving only the participant and 1 or 2 members of the research team (AZ, IMS, and DK). The researchers conducting the interviews had no prior personal or professional relationships with the participants. Before the interviews began, ZDV instructed the researchers on optimal design and conduct of qualitative research interviews and assessed their professional backgrounds, potential biases, and personal interests related to the interview topics. To enhance confirmability, the research team comprised members with diverse theoretical and professional backgrounds, including Sports Sciences, Occupational Therapy, Health Psychology, Nursing, Digital Health, and Clinical Nephrology. Furthermore, team members were actively involved in all stages and levels of the data analysis process. This facilitated critical reflection on emerging interpretations, ensured consideration of multiple perspectives, and helped maintain a close connection between the findings and the participants’ accounts while minimizing the influence of individual researcher biases. All interviews were audio-recorded with the participants’ consent. The interview transcripts were not returned to participants for comments or corrections, and no follow-up interviews were conducted.

### Data Handling and Analysis

During data handling, AZ and IMS transcribed all interview audio recordings verbatim and anonymized them, ensuring the removal of all personal participant data. AZ and IMS manually reviewed all transcriptions, comparing them to the audio recordings to verify the accuracy and authenticity of the transcripts. The authenticated transcripts and audio recordings were subsequently uploaded to the MAXQDA® standard platform (VERBI Software).

We analyzed the collected evidence of health care providers’ perspectives regarding IEG in 2 steps. In the first step, we used Braun and Clarke [66] thematic analysis to inductively explore and identify codes, themes, and their patterns, specifically related to participants’ attitudes and expectations. Two members of the research team (AZ and IMS) repeatedly read the original Slovak transcripts to familiarize themselves with the data (Phase 1) and then independently generated codes for the entire dataset (Phase 2). Quotations included in the paper were translated from Slovak into English by 2 members of the research team (AZ and IMS), and translation accuracy was reviewed by a senior member of the research team (ZDV). Outputs of initial coding were exchanged between 2 members (AZ and IMS) for coding quality cross-check. Four coding meetings (2 online, 2 in-person) were initiated to discuss problematic codes, disagreements in coding, and appeals to recode until consensus in the coding output was achieved. Through iterative discussion and reexamination of the transcripts, codes were refined and, where necessary, recoded until consensus was reached. Both researchers then associated thematically similar codes into groups and subgroups regarding attitudes and expectations on IEG (Phase 3). These themes were subsequently cross-referenced with the raw data to ensure they accurately represented the participants’ experiences (Phase 4). The final output of the coding process and thematic code grouping was reviewed, discussed, validated, and approved by 3 senior members of the research team (JR, ZDV, AMG; Phase 5). At the end of the first step of data analysis, the entire research team documented their findings and collectively summarized health care providers’ attitudes and expectations regarding IEG (Phase 6). Using this procedure, we identified 139 codes and categorized them into 54 subthemes and 5 overarching themes.

In the second step, we used a SWOT analysis approach to organize emerging themes into strengths and weaknesses [potential internal factors], and opportunities and threats [potential external factors]. We conducted a deductive analysis using a revised SWOT model specifically adapted for health care research. The model is built on 3 foundational pillars: addressing stakeholder expectations, evaluating resource management, and considering contextual developments [59]. One member of the research team (AZ) extracted relevant parts from the results of the inductive thematic analysis and categorized them into the 4 quadrants of the SWOT framework. During this process, all codes and subthemes derived from the inductive thematic analysis were systematically examined and categorized as either internal or external factors. Internal factors were defined as those originating within the dialysis centers and directly related to their organizational structures, personnel, operational routines, and implementation processes. External factors were defined as those arising outside the dialysis centers and beyond their direct control, including health care policies, reimbursement frameworks, technological advancements, and broader societal and patient-related influences. They were then evaluated in terms of their hypothetical positive or negative effects on the initiation and implementation processes. Strengths were identified as internal benefits positively related to the IEG at dialysis centers. Weaknesses were identified as internal limitations that might negatively affect or impede the exergaming during dialysis. Opportunities were considered external factors that could positively enhance the applicability and sustainability of IEG. Lastly, threats were identified as external factors that could pose barriers to or adversely affect the implementation of IEG [71]. Afterward, members of the research team (JR, ZDV, AMG; and MA, LK, TJ serving as independent experts not involved in data collection) reviewed and validated whether the content extracted through inductive thematic analysis was appropriate and whether its deductive categorization aligned with the quadrants of the SWOT framework. Using this approach, 43 categories were generated, resulting in 2 strengths, 2 weaknesses, 3 opportunities, and 3 threats regarding IEG. The process of mapping the results of the inductive thematic analysis onto the SWOT framework is presented in the analytical flow table (Multimedia Appendix 2).

### Ethical Considerations

The study protocol was reviewed and approved by the Ethics Committee of the Pavol Jozef Šafárik University in Košice, Slovakia [approval no. 14N/2017]. Written informed consent was obtained from all study participants. We report this qualitative study according to the Standards for Reporting Qualitative Research and the Consolidated Criteria for Reporting Qualitative Research guidelines (Checklist 1) [72,73]. The research team comprised experts from various fields, including Sports Sciences (AZ, PhD), Occupational Therapy (MA, MSc), Health Psychology (IMS, PhD; DK, MSc; ZVD, prof; AMG, prof), Nursing and Digital Health (LK, PhD; TJ, prof), and Clinical Nephrology (JR, PhD and MD). All team members had prior experience in researching older people with chronic diseases, as well as expertise in designing, analyzing, and writing qualitative studies.

## Results

### Characteristics of the Sample (Demographic Data)

We invited 24 health care providers from 2 cooperating dialysis centers to participate in our study. Three care providers (1 nurse and 2 nephrologists) declined to participate due to high workloads and privacy concerns. We conducted semistructured, individual interviews with 3 training assistants, 11 dialysis nurses, 5 nephrologists, and 2 members of dialysis center management. Of the 21 participating health care providers, 11 were recruited from the first dialysis center (6 dialysis nurses, 2 nephrologists, 2 training assistants, and 1 dialysis center manager) and 10 from the second dialysis center (5 dialysis nurses, 3 nephrologists, 1 training assistant, and 1 dialysis center manager). Interviews lasted between 20 and 60 minutes, with an average duration of 39 minutes. One-fourth of the participants were over 50 years old, one-third were male, and half had over 15 years of professional experience. Table 1 provides the demographic and working experience characteristics for each participant’s professional position.

**Table 1.** Participants’ demographic and working experience characteristics by professional position.

Professional position (n)	Sex (male/female), n	Age (years), mean (SD)	Working experience (years), mean (SD)
Dialysis nurse (n=11)	0/11	45.2 (8.1)	18.2 (10.0)
Nephrologist (n=5)	4/1	46.8 (12.6)	20.6 (11.8)
Training assistant (n=3)	2/1	21.7 (0.6)	1.0 (0.0)
Dialysis center manager (n=2)	2/0	55.5 (2.1)	16.0 (1.4)
Total (n=21)	8/13	43.2 (12.5)	16.1 (11.0)

### Overview of Findings From Inductive Thematic Analysis and SWOT Analysis

Using inductive thematic analysis, we identified 139 codes, which we then grouped into 54 subthemes and further organized into 5 overarching themes. These themes reflect a range of perspectives, including (1) mixed attitudes toward IEG: balancing optimism and skepticism, (2) attitudes on the acceptability and long-term sustainability of IEG interventions for patients, (3) expectations for addressing patients’ individual needs in IEG interventions, (4) expected benefits of IEG, and (5) expectations of the potential risks and barriers associated with IEG.

The SWOT analysis led to the identification and formulation of 2 strengths, 2 weaknesses, 3 opportunities, and 3 threats concerning IEG (Figure 1). (1) Strengths included (a) health care providers’ beliefs that exergaming is a safe activity to perform during dialysis, along with their (b) enthusiastic attitudes toward its integration into clinical practice. (2) Weaknesses reflected concerns about (a) the technological complexity of exergaming devices and platforms, and (b) inadequacies in patients’ access to and experiences with digital technologies. (3) Opportunities reported by health care providers included (a) uncertainty regarding alternative forms of exercise that could be implemented during dialysis, (b) calls for changes in dialysis culture, particularly to ensure better availability and greater specificity of information and support for IEG, and (c) suggestions and tips regarding the settings and modifications of exergaming interventions to facilitate their implementation during hemodialysis therapy. (4) Threats included (a) health care providers’ skepticism about the availability of financial resources, (b) factors related to patients’ behavior, personal space, and privacy, and (c) the resistance to IEG interventions among some health care providers. The overall categorization of the outputs derived from the inductive thematic analysis into the SWOT framework is presented in the analytical flow table (Multimedia Appendix 2).

**Figure 1.**
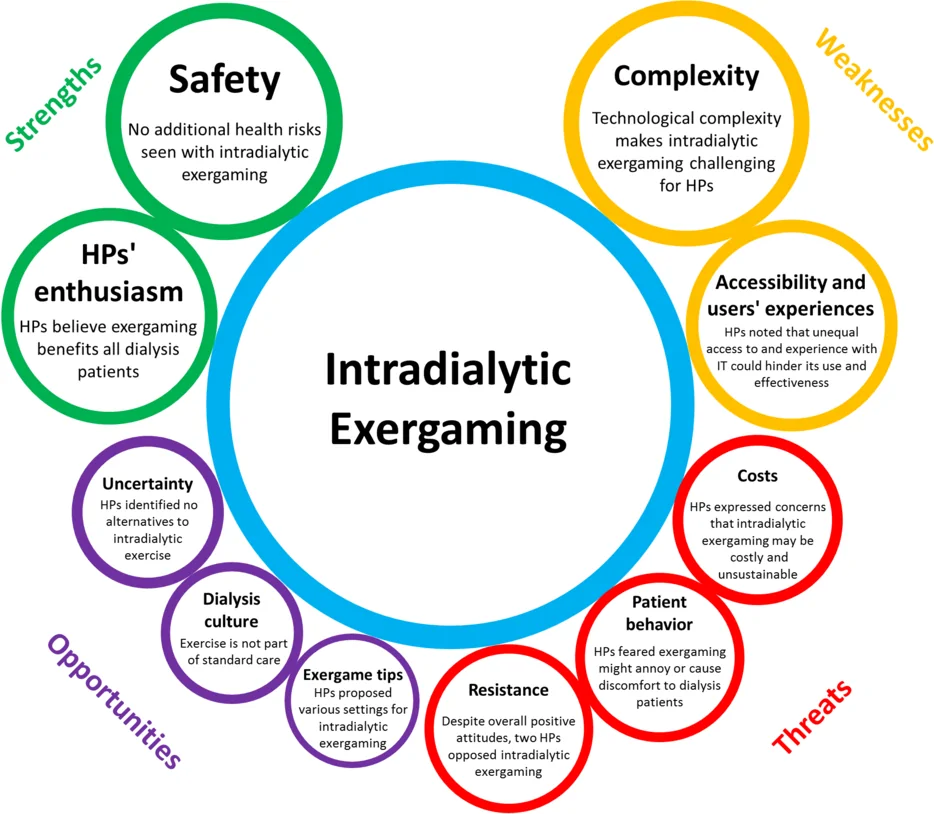
Strengths, Weaknesses, Opportunities and Threats framework (SWOT) analysis template of identified and formulated themes regarding health care providers’ attitudes and expectations on Intradialytic Exergaming (IEG). HP: health care provider.

### Strengths of IEG

#### Safety

Health care providers regarded exergaming as a safe activity to be undertaken during dialysis. They expressed that, when properly introduced, clinically monitored, and technologically supervised, exergaming does not pose any additional health-related risks for patients undergoing hemodialysis. Other participants stated that they were either unaware of or not qualified to comment on any potential health risks that might be affected by IEG.

Health care providers mentioned that they had no prior experience with IEG and limited knowledge and familiarity with technology-based exercise interventions. They anticipated that, prior to the commencement of IEG, both patients and the hemodialysis care team would be adequately introduced to, and familiarized with, the exergaming environment, its functions and controls. Care providers stated that a comprehensive introduction to IEG could help minimize the risks of misuse, underuse, or overuse of the system.

No, I’m not sufficiently educated and experienced in this area [of exergaming]. So, I can’t say exactly what types of exercises are suitable. Because not all stretching or resistance exercise can be done during dialysis… they should definitely be designed by someone who is a muscle [kinesiology] specialist. So, not just like, ‘Here’s a spring or band, exercise with it up and down, and it will go better.

[Nephrologist, male, 44 years old, 20 years of professional experience]

Health care providers emphasized the importance of clinical monitoring in managing potential health-related risks associated with IEG. During hemodialysis, patients must maintain a stable body position to ensure effective extracorporeal blood circulation. Sudden or uncontrolled movements of the torso or limbs could compromise vascular access and impair the efficiency of the dialysis process. Care providers emphasized the necessity of monitoring patients’ activity during IEG to safeguard the adequacy and efficacy of the therapy. They also noted that end-stage kidney disease and the hemodialysis procedure itself may contribute to clinical risk conditions, such as dialysis-induced hypotension, hypertension, dysrhythmias, infections, or bleeding, which could potentially be influenced by exergaming during treatment. As such, these conditions must be closely monitored and evaluated. Once appropriate clinical monitoring is in place, health care providers did not anticipate an increased risk of injury or complications related to the patient’s clinical profile.

We have quite an aged dialysis population. So, they generally have quite osteoporotic bones. It really needs to be individualized based on who the patient is, right? Because we have elderly women who have recently fallen. So, it’s really necessary to approach each patient individually, considering their diagnoses, their age, and essentially what can be done with them, just like you were assessing at the beginning, I believe.

[Nephrologist, male, 40 years old, 10 years of professional experience]

The safety risk could be that the patient becomes so immersed in the game that they forget they are connected to the dialysis machine. They might get agitated during gameplay, causing their blood pressure to rise, or they could accidentally pull out the needle with a sudden movement or if they get startled. Such consequences would need to be addressed.

[Nephrologist, male, 40 years old, 10 years of professional experience]

No [risks for IEG], I don’t think so. Only if it could be that his [patient’s] blood pressure increases [during exergaming], right? And then the consequences would have to be dealt with.

[Nurse, female, 38 years old, 16 years of professional experience]

Regular inspections of exergaming technology used in dialysis health care settings are essential to ensure patient safety. Health care providers noted that undetected defects, faults, or failures in exergaming systems could increase the risk of injury and health-related complications. However, they stated that these risks could be mitigated through regular, properly conducted technical inspections, including evaluations of both the physical integrity and functional safety of the exergaming technology.

I would say the technical aspect of such things [exergaming]. It is related to some devices or controllers, and it is electronics, you know, which is not that simple. Technical side of dialysis is my area and everything technical here requires certain maintenance and also inspection when used. Within a certain time frame during use. All of this must be well controlled.

[Manager, male, 54 years old, 9 years of professional experience]

The question is, who is supposed to technologically secure all of this, the technology. We were distributing headphones to patients for listening to the television program… they got damaged.

[Nephrologist, 68 years old, 40 years of professional experience]

#### Health Care Providers’ Enthusiasm

Patients undergoing hemodialysis often spend treatment sessions engaging in passive activities such as watching television, browsing the internet, reading, or listening to music. However, health care providers have noted that these activities often become monotonous and repetitive, leaving patients feeling bored, as they are disconnected from their real-life interests and hobbies. Care providers anticipated that IEG could serve as a more stimulating and varied alternative, an unconventional activity that captures attention, is personalized, and offers greater enjoyment through content that is engaging, distracting, and fun.

It might be fun and maybe the dialysis would pass more quickly for them... That would be perfect because they would have visuals, maybe play something, and just exercise along with it. Would that be one way to motivate the patient? Even someone who is shy might get into it.

[Nephrologist, male, 43 years old, 19 years of professional experience]

Yes, because many of them watch things that are completely pointless or repetitive. There are people, yes, I can say, there are people here who watch educational programs, which is great. They are constantly learning. But there are also younger ones, for example, a 30-year-old who brings a series and basically watches it for 4 hours, which I think he doesn’t need to do, he could be doing something else. He’s too young for this.

[Nurse, female, 38 years old, 16 years of professional experience]

### Weaknesses of IEG

#### Complexity

Participants identified the technological complexity of using exergaming devices and platforms in clinical settings as a significant limitation. Several noted that limited space in some dialysis centers could hinder the installation and positioning of such equipment. They emphasized the importance of maintaining unobstructed access around the patient, particularly the area between the patient’s vascular access site and the dialysis machine. Regarding design and installation setup, health care providers expressed a preference for exergaming devices and platforms to be wireless and equipped with internal or built-in power supplies. For positioning, they favored designs that could be securely attached to the external structure of the dialysis bed using a fastening system, allowing for quick and easy detachment by health care staff when necessary.

Because those beds are really designed for the patient to fit in, and maybe have something next to them. So, depending on how big the device would be, I’m afraid it would be a bit restrictive in terms of placement, right?

[Nephrologist, male, 45 years old, 16 years of professional experience]

#### Accessibility and Users’ Experiences

With regard to other limitations, health care providers expressed 2 broad groups of factors that may influence patients’ engagement with IEG. The first group relates to technological accessibility and digital experience. Participants noted that patients undergoing hemodialysis, particularly those with limited access to digital technologies and minimal previous experience using them in daily life, may be skeptical, dismissive, or uninterested in IEG. They also noted that socioeconomic background and prior digital experience, particularly the frequency and quality of that experience, were strongly associated with patients’ capacity to accept and adopt digital technologies in health care settings. The second group comprises individual patients’ characteristics affecting technology use. Participants identified impaired visual, auditory, and cognitive functions as significant barriers to engaging with digital technologies, potentially hindering patients’ ability to participate in exergaming during dialysis sessions. Interestingly, there was inconsistency among care providers regarding the role of age in patients’ acceptance of exergaming technologies. Some mentioned that IEG is unsuitable for older patients undergoing hemodialysis, while others perceived no age-related disadvantages. The remaining participants acknowledged that older age could present a barrier but emphasized that many older patients are fully capable of using and benefiting from digital tools.

In my opinion, there are older patients who will find it harder to handle [exergaming device/platform] than a mobile phone. So, this will be more difficult for them. But sometimes we are surprised that even an 80-year-old grandmother has her [own] phone and can use it well. So, it would probably need to be assessed individually first. I can’t say definitively yes or no for everyone.

[Nephrologist, female, 34 years old, 8 years of professional experience]

Well, we have a computer available for patients if they are interested. We have internet in the hall [dialysis room] if patients are interested, but almost no one used it.... But I don’t know if it would work like ‘here you go, play.’ And of course, they have to be... physically and mentally fit to do something like that [IEG].

[Nurse, female, 54 years old, 36 years of professional experience]

### Opportunities of IEG

#### Uncertainty

Most of the health care providers were unable to suggest specific types and settings that could be used to tailor exergaming interventions to better suit the clinical environment, as well as the needs, expectations, and limitations of both health care providers and patients undergoing hemodialysis. The limited ability of health care providers to identify external modalities for tailoring exergaming interventions to the hemodialysis setting indicated an opportunity for future research, technological innovation, and stakeholder engagement to support the implementation of IEG. To enhance the usage of intradialytic exercise and exergaming, care providers identified several opportunities: increasing health care providers’ awareness and knowledge related to the planning and organization of such interventions; ensuring either permanent or temporary access to exercise specialists for patients undergoing hemodialysis; and fostering international networks to support the exchange of best practices and shared experiences in this field.

You are asking me a difficult question, and I don’t know, at the moment I don’t know, I haven’t thought about it [variations of intradialytic exercise], what and it would probably be the same with everyone [of participants]… because basically, when we think about it, we are always dealing only with the patient’s health condition.

[Nurse, female, 38 years old, 16 years of professional experience]

#### Dialysis Culture

Intradialytic exercise is not currently integrated into routine hemodialysis care in Slovakia. According to health care providers, exercise during dialysis, regardless of its form, is not recognized as a standard component of health care services. They reasoned that the health care system, particularly its legislative and organizational frameworks, has not yet been adapted to support the integration of such services into clinical practice. Drawing on experiences shared by participants during their visits to dialysis centers abroad, they provided suggestions and proposed strategies for implementing effective IEG interventions. Opportunities to enhance the use of IEG include changes in dialysis culture, including creating a more family-like and relaxed clinical environment within dialysis centers; providing direct legislative support for such activities; and establishing complementary services such as nutritional and psychological counseling in routine hemodialysis care.

But this is not just a local problem of our [clinical] workplace; it is a systemic error. Simply put, the entire healthcare system [in Slovakia]. So we cannot sustain [nutrition status among] patients and do not exercise with them… In the [other areas of the] world, it works exactly like that: they know how many kilojoules a patient needs, what kind of diet they need, and which day after acute treatment they can start exercising.

[Nephrologist, female, 34 years old, 8 years of professional experience]

I can’t imagine it [routine intradialytic exercise]. Because, for example, when you look at dialysis abroad, they dance and exercise with the patients, and there’s a more relaxed atmosphere. Here, it’s just so clinical, the dialysis, connecting and disconnecting, needles, everything sterile. It seems to me that everything has its rules, everything must be on time.

[Nurse, female, 34 years old, 10 years of professional experience]

#### Exergame Tips

After receiving a verbal introduction to intratherapeutic exercise interventions from members of the research team during the interview, health care providers were able to offer several suggestions regarding the setting and potential variations for implementing IEG. They proposed that patients could engage in endurance exercises such as bed bike pedaling, resistance exercises using rehabilitation equipment, and relaxation or breathing exercises during dialysis. According to them, these activities could be delivered in the form of exergaming and might be enhanced with accompanying music. Other participants suggested incorporating exergaming as a routine component of health care but recommended scheduling exergaming sessions at dialysis centers immediately after the completion of dialysis treatments, rather than during the sessions themselves.

Maybe they could actually pedal on those bikes, for example. I don’t mean the ones used by athletes, but there are some, I don’t know what they’re called, where the patient sits upright, and they’re actually used during rehabilitation.

[Training assistant, male, 23 years old, 1 year of professional experience]

### Threats of IEG

#### Costs

Health care providers expressed uncertainty when identifying the entity or authority that might be responsible for funding IEG services. They expressed skepticism about the applicability and long-term sustainability of such interventions within the Slovak health care system, primarily due to the anticipated high initial financial investment and limited funding opportunities for supplementary health services. Several participants were unwilling to discuss or chose not to clearly articulate their views on the financial aspects of implementing and sustaining IEG. One participant suggested that demonstrating both the health-related benefits for patients and the cost-effectiveness of the intervention could serve as a foundation for further discussions with health care management organizations and stakeholders about securing additional financial support for such intratherapeutic services.

Well, for them it might actually be fun, and maybe it would make the dialysis go by faster for them, but who would fund it? Yeah, like for him [patient] it might actually be [fun], yeah, just unfortunately, who would fund it, who would take care of it?

[Nephrologist, male, 43 years old, 19 years of professional experience]

#### Patient Behavior

The health care providers expressed the view that due to the presence of kidney diseases and the complexity of associated health complications, patients often experience periods of frustration, demotivation, resignation, negativism, and oppositional behavior. Furthermore, care providers who participated in our study mentioned that each patient presents a unique set of personality traits, with an individual trajectory of development and expression. As a result, care providers anticipated that the introduction, familiarization, and adoption of the IEG concept would require a long-term, sustained effort and a personalized approach. They recommended avoiding strict, formal, task-oriented communication, and instead emphasized the importance of a sensitive, familiar, and pressure-free approach during the introduction and initiation of IEG. With regard to the specific nature of exergaming, health care providers mentioned that some patients might feel embarrassed due to the visibility of the activity within the shared space of dialysis rooms, as well as unfamiliarity with the exergaming technology among patients. Two participants specifically noted that differences in levels of game immersion could influence patients’ engagement with exergaming. They also emphasized that psychological predispositions toward gaming addiction should be considered a potential risk associated with IEG.

Health care providers expressed concerns that patient behavior-related factors, which are largely external to the intervention and beyond the direct control of health care providers, could significantly influence patients’ attitudes and willingness to engage with exergaming during hemodialysis therapy, and, as a consequence, could threaten the acceptability, usability, and sustainability of IEG.

As for barriers [for exergaming], what comes to my mind is addiction. Because after all, these [exergaming interventions] are games, and addiction to games is quite easy to develop, so from that perspective.

[Training assistant, female, 22 years old, 1 years of professional experience]

With regard to other potential risks, participants noted that violations of patients’ personal space, privacy, and comfort during dialysis caused by exergaming activity could compromise the usage of intradialytic exercise interventions. Such disruptions could also affect patients who are not directly participating in exergaming during their treatment. Health care providers identified several sources of these intrusions, including changes to the timing of dialysis connection and disconnection procedures, alterations to the spatial layout of the clinical environment during hemodialysis, and increased noise levels or other external factors that may be perceived by patients as negative distractions during therapy.

I think these are some patients who... how to put it? They’re kind of offended by the whole world, and they would seriously cause problems and refuse [exergaming], and maybe they would even cause problems for the other patients [whose exergaming during their dialysis] if they didn’t feel comfortable in some way, and if it somehow limited them. I mean the exercise of the other patients, not their own.

[Training assistant, male, 21 years old, 1 year of professional experience]

If there were such interest, then the conditions would have to be created for it [IEG]. Because we have a large hall [dialysis room]. There are a lot of people there. Right? The question is how the others would react, whether it [exergaming] would disturb them.

[Nurse, female, 54 years old, 36 years of professional experience]

#### Resistance

We identified critical, negative, and mixed attitudes and expectations regarding IEG, including resistance to the use of digital tools. Two health care providers expressed strong opposition to the integration of exergaming into dialysis therapy. They expressed a sense of technological resistance and frustration, or overwhelm, with the increasing reliance on digital tools. In the context of intradialytic exercise, they demonstrated a clear preference for traditional, patient-centered approaches to health care, placing high value on direct, personal interaction over technology-mediated experiences. Additionally, 2 other caregivers anticipated initial resistance to IEG and raised concerns about the added responsibilities it could place on health care providers, as well as potential disruptions to the routines of dialysis nurses.

Well, I already think this is a bit over the top with these computers. Everyone should maybe step away from it a little, I would say. You know, it’s [exergaming] not the same as when you’re physically with the patient. It’s not that kind of contact. It’s just the computer again, and then some pedals again.

[Nurse, female, 60 years old, 40 years of professional experience]

I would probably be lying if I said it [IEG] would go smoothly. I’m expecting initial resistance, definitely. [It means] A load of additional work [for healthcare providers]. That’s just how it is. A burden, simply because it’s another [work]load.

[Nephrologist, female, 34 years old, 8 years of professional experience]

## Discussion

### Principal Findings

This study aimed to explore the attitudes and expectations of health care professionals regarding the concept of IEG as a strategy to support patient self-management in the domain of physical activity. It offers novel insights into the perceived strengths, weaknesses, opportunities, and threats associated with IEG, as reported by training assistants, nurses, nephrologists, and dialysis center managers.

Health care providers identified the perceived safety of IEG, along with generally positive and enthusiastic attitudes toward its use, as key strengths. However, they also highlighted the complexity of the exergaming concept, as well as closely related issues of accessibility and user experience, as potential weaknesses that could hinder its integration into health care services. In terms of opportunities, participants expressed uncertainty regarding alternative approaches to the organization and delivery of intradialytic exercise. They emphasized the need for cultural adjustments within dialysis settings to facilitate the implementation of various proposed IEG modalities in the clinical environment. The main threats identified included skepticism about the financial and resource implications of IEG, potential patient reluctance or refusal to participate due to behavioral factors, and resistance to change among health care professionals in adopting exergaming as part of routine care.

Patients’ perceptions of safety play a key role in their decision to initiate and continue using technology-based physical activity interventions during dialysis therapy [74,75]. Our findings regarding perceived strengths suggest that health care providers generally view exergaming as a safe activity for patients undergoing hemodialysis. This aligns with existing evidence from studies monitoring patient safety, which consistently report no serious adverse events attributable to IEG [42,76-80]. From a broader perspective, research on exergaming interventions in patients with heart failure, stroke, or cancer similarly reports either no adverse events or only rare, exceptional cases [81-83]. Paradoxically, evidence from older adults and patients with multiple sclerosis, mild cognitive impairment, other neurocognitive disorders, and hypertension suggests that exergaming may raise concerns about physical safety. These concerns include fear of falls and general injuries, as well as occurrences of knee or back pain [16,84-89]. Such safety issues have been attributed to reduced awareness of the physical environment and insufficient guidance and support during exergaming activity [84,85,90]. Our participants expressed that, when properly introduced, clinically monitored, and technologically supported, IEG does not pose additional health risks to patients. They also expressed enthusiastic attitudes toward its use during hemodialysis therapy, which is consistent with existing evidence on positive perceptions among health care providers working with patients with dementia, cardiovascular conditions, stroke, and cancer [53,55,82,91,92]. Taken together, these insights into perceived strengths underscore the importance of promoting IEG by prioritizing patient safety and leveraging the enthusiastic support of health care providers. Their positive perceptions can play a pivotal role in enhancing patient engagement and long-term adherence to technology-supported physical activity during dialysis therapy.

Technological limitations and a lack of design flexibility in exergaming devices and platforms may undermine their ecological validity and hinder their transferability into clinical practice [93-95]. Our participants identified several weaknesses related to the design, installation setup, and positioning of exergaming technologies within dialysis environments, collectively referred to as technological complexities. These included limited space in dialysis centers, the need to maintain unobstructed access between the patient and the dialysis machine, and preferences for wireless, self-powered devices that can be securely yet easily attached to dialysis beds. These findings are consistent with existing evidence on the general limitations and challenges of exergaming among patients undergoing hemodialysis, particularly those arising from environmental and organizational constraints [96-99]. Importantly, other authors have noted that such complexities may present challenges not only during the integration of these technologies into clinical settings but also for health care providers and end-users [55,100-105]. Our participants also identified additional barriers to IEG, including patients’ digital literacy, sensory and cognitive impairments, socioeconomic factors, and differing views on the influence of age on user experience. These factors were seen as key challenges that may limit patients’ accessibility and engagement with exergaming technologies [16,56,97,98,100,101,103,106]. To improve the quality of implementation, particularly the transferability and accessibility of IEG technologies in clinical practice, developers and promoters should be aware of and take into account the specific conditions of dialysis environments and patient-related limitations from the earliest stages of design and development.

Health care providers are central figures in the development, initiation, and long-term sustainability of innovative therapeutic approaches in clinical practice [107,108]. In relation to opportunities for IEG, participants expressed limited ability to tailor such interventions to clinical settings and individual patient needs, highlighting gaps in their awareness, knowledge, and experience. Nevertheless, they proposed several key opportunities to enhance implementation, including raising awareness among health care professionals, integrating exercise specialists into routine dialysis care, and fostering international collaboration to share best practices. Although intradialytic exercise is not yet a standard component of dialysis care in Slovakia, participants suggested feasible strategies to support its integration. These included legislative backing, cultural shifts within dialysis centers, and the availability of complementary services. Our evidence on health care providers’ expectations and proposed opportunities aligns with existing literature, which emphasizes the importance of health care providers’ competencies, the presence of support teams and services, and the need for legislative and leadership support within dialysis networks [107,109-114]. Enhancing the availability of professional competencies and providing institutional and legislative support may serve as foundational drivers for cultural change in dialysis care. Such improvements could help meet the needs and expectations of health care providers and enable broader implementation of IEG in clinical practice. A recent study highlights that implementation of innovative exercise interventions, such as exergaming, depends on addressing multilevel factors, such as patient needs, health care professional engagement, and supportive policy frameworks [105].

Underestimating external factors, particularly potential threats, during the planning of behavioral interventions can compromise their usage in clinical practice. Our participants expressed uncertainty and skepticism regarding the financial feasibility of IEG, emphasizing the need for robust evidence of its health benefits and cost-effectiveness. Patient behavior was also identified as a potential threat, with participants highlighting the importance of a personalized and sensitive approach to introducing exergaming, given the psychological, social, and environmental challenges that may influence initial engagement and long-term adherence. Additionally, some health care providers expressed resistance to the concept of IEG, favoring traditional care methods and raising concerns about technological burden, workflow disruptions in dialysis settings, and potential patient discomfort. Existing literature reflects similar concerns, with reports of uncertainty and skepticism about the availability of financial resources for exergaming [89,92,115,116], although other studies highlight its favorable cost-effectiveness and cost-efficiency [117-119]. However, cost analyses specific to IEG are currently lacking and are critically needed to inform planning and development. Patient behavior, particularly motivation, and social and emotional factors has been significantly associated with the effectiveness and adherence to exergaming interventions across diverse populations, and is recognized as both a key determinant and a potential threat [120-124]. A particularly salient concern raised by health care providers was the potential for gaming addiction, reflecting a critical awareness of unintended psychological consequences that may arise from IEG. The evidence highlighting resistant, reserved, and oppositional attitudes, as well as expectations surrounding IEG, represents a challenging yet essential insight for effective planning and development. Recommendations to overcome initial resistance include the provision of education and support from specialized professionals (eg, physiotherapists), and the active involvement of health care providers in the planning and management of the intervention [65,109,113,125]. To effectively mitigate potential risks, it is essential to conduct a thorough assessment of the resources required, prioritize individualization by addressing each patient’s specific needs, and actively involve health care providers in the planning process prior to implementation of IEG.

Our study provides comprehensive evidence on Slovak health care providers’ attitudes and expectations regarding the implementation of IEG in clinical practice, systematically organized into thematic quadrants of the SWOT framework. Future research exploring perceptions of IEG, including those of patients, exergame developers, and stakeholders (particularly patients’ organizations, governmental and regulatory bodies), could enhance understanding of critical factors for strategic planning. Evidence on attitudes and expectations toward IEG from respondents representing diverse health care systems, geographical regions, and socioeconomic and cultural backgrounds could strengthen the generalizability of these findings.

This study presents several key strengths. First, the sample included professionals from all levels of the organizational hierarchy within dialysis centers, from training assistants and nurses to nephrologists and senior management, providing a well-rounded and comprehensive perspective on health care providers’ attitudes and expectations regarding IEG. Second, the use of prescreening enabled the recruitment of a highly diverse group of participants, varying in age, gender, and professional background. Third, all participants contributed relevant expertise through their direct involvement in implementing intradialytic exercise interventions in clinical practice. Fourth, we used an inductive approach to analyze health care providers’ attitudes and expectations, followed by deductive categorization using the SWOT framework. We believe this methodological strategy yielded a practical, systematically organized, and transferable overview of the SWOT associated with IEG interventions, offering valuable insights to support strategic planning and informed decision-making.

This study has several limitations that should be acknowledged. First, despite our efforts to ensure a balanced representation across professional roles, we were unable to achieve the optimal sample size of 6 to 8 participants in the groups representing dialysis center managers and training assistants. However, the strong thematic alignment between managers, training assistants, and other professional groups suggests that their perspectives were largely reflected. Second, all participants were recruited from 2 dialysis centers affiliated with leading nephrology networks in Slovakia. As a result, the findings may be shaped by context-specific factors such as socioeconomic, demographic, and cultural characteristics, which could limit their generalizability to other dialysis settings with different health care structures. Third, our inclusion criteria required varying minimum levels of professional experience across roles. This decision was made to address the limited availability of experienced training assistants and to avoid potential bias from insufficiently experienced nurses, nephrologists, and managers. Nonetheless, this approach may have excluded early-career perspectives from some professional groups. Fourthly, our study is limited by the fact that participating health care providers had no direct experience with IEG. Therefore, the findings are based on hypothetical perceptions and expectations regarding its implementation in hemodialysis care and may not fully reflect experiences or challenges associated with its actual use in clinical settings.

### Conclusions

This study offers valuable insights into health care providers’ views on the implementation of IEG in clinical practice. They regarded IEG as safe and potentially beneficial for patients undergoing hemodialysis when supported by clinical monitoring and technological oversight. They highlighted the potential of IEG to enhance patient engagement and alleviate boredom during hemodialysis while noting weaknesses such as technological complexity and limitations on accessibility and users’ experiences. Health care providers identified opportunities such as clarifying alternative exergame options, enhancing the availability and quality of information and support for IEG, and refining available IEG settings and modifications to facilitate implementation during hemodialysis therapy.

Although IEG is not yet standard practice in Slovakia, our findings indicate that, with appropriate planning, training, and policy adjustments, it could become a sustainable component of hemodialysis care. Health care providers acknowledged its potential benefits for engagement and well-being but raised concerns about financial feasibility, long-term sustainability, and availability of support for integration into the health care system. Common threats included uncertainty over funding and skepticism about health care system readiness, alongside worries about patients’ privacy, clinical routines, staff workload, and resistance. In addition, our participants emphasized the need for a personalized approach to address patients’ psychological variability and possible discomfort in shared spaces. The findings highlight the need to address both human and systemic factors in the design, development, and implementation of IEG, indicating that successful integration will require not only technological innovation but also cultural, organizational, and legislative support.

Health care providers regarded IEG as a safe and promising option for patients on hemodialysis, particularly for boosting engagement and reducing treatment-related boredom. To make this approach feasible in practice, clear guidelines, staff training, and organizational support are essential to overcome technological and accessibility challenges. With thoughtful planning and adequate resources, IEG could become an effective addition to hemodialysis care, improving patient experience and overall well-being.

## Supplementary material

10.2196/106283 Multimedia Appendix 1 Interview guide used in this study.

10.2196/106283 Multimedia Appendix 2 Analytical flow table for mapping and categorizing inductive thematic analysis findings within the Strengths, Weaknesses, Opportunities, and Threat (SWOT) framework.

10.2196/106283 Checklist 1 Consolidated Criteria for Reporting Qualitative Research (COREQ) checklist

## Abbreviations

IEG: intradialytic exergaming
SWOT: Strengths, Weaknesses, Opportunities, and Threats
