# Supplementary material for: Analysis of Health Care Providers' Attitudes and Expectations on Intradialytic Exergaming: Qualitative SWOT Study
Source: JMIR Rehabil Assist Technol. 2026 Nov 4;13:e106283. doi: 10.2196/106283 (PMC13734701; doi:10.2196/106283)
Supplement: Supplementary file 1 [file rehab-v13-e106283-s001.docx]

**Multimedia Appendix 1	Interview Guide**

**1^st^ Scope area**

***Benefits of physical activities for dialysis patients in general***

**Main question:	What are the benefits of physical activities for dialysis patients?**

**Sub-questions:**

1. Do you think that physical activities are suitable for dialysis patients? Which benefits could physical activity induce/bring? (health, psychological, social, economic impacts)
2. What risks could be associated with physical activity in dialysis patients?
3. Why do you think some patients prefer a sedentary rather than an active lifestyle? What reasons can be given for this phenomenon?

**2^nd^ SCOPE AREA**

***BENEFITS AND RISKS OF INTRADIALYTIC EXERCISE***

**Main question:	Do you consider a patient’s exercising during dialysis to be appropriate? Can you justify your opinion?**

**Sub-questions:**

1. Can patients benefit from exercise during dialysis? (health, psychological, social, economic benefits)
2. What risks could be associated with exercise during dialysis?
3. Do you think that exercise during dialysis could improve patients’ quality of performing habitual daily activities?
4. Can exercise during dialysis help patients better cope with dialysis therapy?

**3^rd^ SCOPE AREA**

***IMPLEMENTATION OF EXERCISE INTERVENTIONS IN CLINICAL SETTINGS***

**Main question:	What was your experience with the implementation of intradialytic exercise in the dialysis unit?**

**Sub-questions:**

1. What factors could aggravate the implementation of such exercise interventions in a dialysis unit? (from a patient’s, different care-providing personnel, management, point of view etc.)
2. What do you think could motivate patients to exercise during dialysis?
3. Who should implement an intervention in a dialysis unit? Who should finance and support such an intervention? Who are the “key players” in the successful implementation of such an intervention?
4. If patients exercise during dialysis routinely, do you think it would add to the workload of the care-providing staff? What is your opinion about routine intradialytic exercise and the extent (level) of acceptability for such an intervention among care-providing staff?
5. Are you comfortable counselling patients about exercise? Do you have any training in this area? If yes, was your training sufficient for your counselling activities with patients? If not, what would help make you feel more comfortable during counselling activities?
6. Which patients should be recommended for exercise during dialysis? Which patients should be involved?
7. Why do some patients declined intradialytic exercising even when it is offered to them?
8. Do you think there is enough time and space in dialysis care to perform exercise during haemodialysis?
9. Do you think there is enough care-providing staff present in the dialysis unit to carry out such exercise interventions without affecting the dialysis therapy operations?

**4^th^ SCOPE AREA**

***VARIATIONS IN INTRADIALYTIC EXERCISE INTERVENTIONS***

**Main question:	Can you imagine and explain what (other) form of exercise would be possible to implement at your dialysis unit?**

1. Can you imagine the implementation of exercise during dialysis by computer games or exergaming?
2. Would patients welcome such diversification of the time spent at the dialysis therapy?
3. Can you identify certain barriers or risks for patients in this approach?
